# Diagnostic Perspectives on the Relationship Between Paraspinal Muscles and Bone Mineral Density: A Narrative Review

**DOI:** 10.3390/diagnostics16132010

**Published:** 2026-06-27

**Authors:** Hui Liu, Moran Suo, Sinuo Yan, Qiwen Wang, Xin Chen, Zhonghai Li, Chunli Zhang

**Affiliations:** 1 Emergency Department, Seventh Medical Center of PLA General Hospital, Beijing 100162, China; dyyang2003@126.com; 2 Department of Orthopedics, First Affiliated Hospital of Dalian Medical University, Dalian 116600, China; ssuomoran@163.com (M.S.); 15004007306@163.com (S.Y.); wangqiwen20011016@163.com (Q.W.); benjaminchen@link.cuhk.edu.hk (X.C.); 3 Department of Orthopedics, Fourth Medical Center of PLA General Hospital, Beijing 100048, China

**Keywords:** paraspinal muscles, bone density, osteoporosis, sarcopenia, aging

## Abstract

**Background:** With the global population aging, osteoporosis and associated vertebral compression fractures are increasingly prevalent. While bone mineral density (BMD) remains the standard clinical parameter for diagnosing osteoporosis, it provides an incomplete assessment of holistic spinal bone health. Paraspinal muscles are essential for spinal stability and movement. This narrative review aims to evaluate the interplay between paraspinal muscle status and lumbar spine BMD, thereby providing a scientific foundation for comprehensive bone health management. **Methods:** A comprehensive literature search was conducted in PubMed using relevant keywords to identify studies evaluating the functional and morphological interactions between paraspinal muscles and bone mineral density. **Discussion:** Current evidence demonstrates a significant negative correlation between paraspinal muscle FI and lumbar spine BMD. High FI is identified as an independent risk factor for osteoporotic vertebral fractures and a robust predictor of postoperative complications. The relationship between paraspinal muscle CSA and BMD remains debated. Nevertheless, targeted high-impact and resistance training generate substantial mechanical loading through muscle contraction, providing biological stimuli for trabecular BMD preservation. **Conclusions:** Evidence highlights a critical synergy between paraspinal muscle status and bone mineral density, with muscle fat infiltration acting as a key marker for bone loss and fracture susceptibility. Integrating these muscle parameters with traditional BMD measurements improves fracture risk stratification and osteoporosis management.

## 1. Introduction

With the global aging population trend, the incidence of musculoskeletal diseases such as osteoporosis continues to rise [1]. With advancing age, increased brittleness is noted in bone, and the mechanical properties of both cortical and trabecular bone gradually decline [2,3,4]. Spinal aging is characterized by degeneration of vertebral structure and paraspinal muscles. Osteoporotic compression fractures are very common in the elderly population, which often leads to persistent pain in the lower back of patients. Osteoporosis is defined as a systemic skeletal disorder characterized by low bone mass and microstructural deterioration of bone tissue, resulting in increased bone fragility and susceptibility to fractures [1]. Because of the systemic nature of osteoporosis, the increased risk of fractures affects nearly all skeletal sites [5]. Even within the elderly population, older individuals still exhibit a higher incidence of fractures [6]. Osteoporosis is the most common osteometabolic disease, characterized by a reduction in bone density and microarchitectural deterioration. Fragility fractures are the main complication in osteoporosis, with a global increase in fracture risk. Osteoporosis represents a significant public health challenge, with an estimated annual cost of $34.8 billion worldwide. With projected demographic changes, this burden is expected to increase [7].

Vertebral fractures have a close association with reduced vertebral bone mineral density (BMD) and have historically been considered typical of osteoporotic fractures [8]. Vertebral fractures are the most common fractures associated with low BMD and represent a significant source of morbidity, mortality, and other adverse health outcomes [9]. Reduced BMD is an important manifestation of bone aging. The reduction in bone mechanical properties is associated with increasing age. The increase in age affects all parameters except for elastic stiffness in bone [10]. BMD measurements have been compared across various species using various techniques, indicating an increase in bone mineral content during growth and development, followed by a decline in the later stages of aging [11,12,13,14]. However, solely assessing bone aging through BMD is incomplete. When compared to younger individuals with similar BMD, older individuals may experience a tenfold increase in the 10-year risk of fractures [15]. Nevertheless, BMD remains a commonly used parameter in clinical settings to assess the risk of fractures. Many studies have shown that the BMD of several skeletal sites can predict vertebral fractures, but in most research, lumbar spine BMD is found to have a stronger correlation with vertebral fractures compared to BMD at other skeletal sites [16,17,18].

Paraspinal muscles, the muscle groups surrounding the spine, play a vital role in supporting the spine, maintaining body posture, and facilitating spinal movement. In recent years, a significant body of research has shown a complex and intricate relationship between paraspinal muscles and various spinal conditions [19]. Changes in paraspinal muscle cross-sectional area (CSA), fat infiltration (FI), and functional status are often associated with lower back pain, lumbar disk degeneration, and other spinal issues [20,21,22]. In the elderly population, lower CSA and a higher FI can be observed in the paraspinal muscles [23].

Osteoporosis is often diagnosed only after the occurrence of the first clinical fracture, since bone loss is a silent and initially asymptomatic process. Therefore, it is crucial to assess an individual’s osteoporosis risk early on to prevent initial fractures [24]. There is an urgent need to establish a more comprehensive assessment system for the evaluation of spinal aging for clinical practice. Through the comprehensive literature analysis and the latest research data, this review will explore the relationship between paraspinal muscle functional status and BMD, aiming to provide a better understanding of the interaction between BMD and paraspinal muscles, offering a scientific foundation for holistic bone health management and predicting aging.

## 2. Literature Search Strategy

To ensure a comprehensive evaluation of the topic, a literature search was conducted using the PubMed database. The primary search strategy utilized the Boolean keyword combination: “paraspinal muscles” AND “bone mineral density”. To capture the most recent and relevant evidence as requested during the peer-review process, the search timeframe was defined from the database’s inception up to April 2026. Articles were included if they evaluated the morphological or functional relationships between paraspinal muscles and spinal bone health in human subjects. Non-English publications and studies focusing exclusively on appendicular musculature without assessing the lumbar spine were excluded from this narrative review.

## 3. Bone Mineral Density

The average measurement of BMD within vertebral bodies is frequently utilized as a predictive indicator for vertebral strength and fracture risk [25]. While Dual-Energy X-ray Absorptiometry (DXA) evaluating areal BMD remains the diagnostic gold standard for osteoporosis, quantitative computed tomography (QCT) evaluating volumetric BMD offers a comparable and more sensitive method for detecting bone mineral loss [26]. Clinically, lumbar spine BMD exhibits a significant decrease with age, along with declines in weight and fat mass. Trabecular volumetric BMD starts to decline linearly before reaching middle age, accounting for substantial trabecular bone loss over the lifespan in both sexes. Accelerated bone loss is characteristically observed in the early postmenopausal period in females, where decreasing lean mass exerts a significant influence on lumbar BMD. Ultimately, relying solely on BMD to assess bone aging is incomplete; therefore, complementary imaging biomarkers are required for a more precise evaluation of fracture risk in elderly populations.

Lumbar spine BMD exhibited a significant decrease with age, along with declines in weight and fat mass [27]. Trabecular volumetric BMD starts to decline before reaching middle age, following a linear trajectory [28]. While conventional wisdom suggests that bone loss begins at menopause in women and later in life in men, there is substantial trabecular bone loss in both sexes during young adult life. This accounts for between one third and one half of the total trabecular bone loss over the lifespan in both sexes [29]. In the elderly population, lumbar spine BMD in males is significantly higher than in females, and both exhibit age-related declines [30]. Accelerated bone loss is observed in the early postmenopausal period in females, with the rate of decline in BMD slowing down as the postmenopausal duration extends [31,32]. Body mass index and lean mass decrease with aging, exerting a significant influence on lumbar BMD in early postmenopausal women [33]. A meta-analysis showed the increase in BMD was notably lower with advancing age in the spine in individuals treated with parathyroid hormone [34].

## 4. Paraspinal Muscles

### 4.1. Paraspinal Muscle Cross-Sectional Area and Fat Infiltration

Paraspinal muscles are a group of muscles located on either side of the spine and play a crucial role in the stability and function of the spine. The morphology of paraspinal muscles can be evaluated through various imaging techniques, such as CT and MRI. Commonly used imaging parameters include CSA and FI. The CSA signifies the magnitude or mass of the musculature, whereas FI conveys insights into the adipose content embedded within the muscular tissue. There are three methods for assessing FI: visual qualitative assessment, semiquantitative assessment, and quantitative assessment. Among these, semiquantitative and quantitative assessments are commonly employed in research to evaluate FI [35,36,37].

Paraspinal muscles play a crucial role in the stability and function of the spine, and their morphology is typically evaluated via CT and MRI. The CSA signifies muscle mass, whereas FI conveys the adipose content embedded within the muscular tissue. Clinically, a decline in CSA and an increase in FI are hallmark indicators of muscle degeneration, reflecting lower muscle quality and diminished spinal function [38,39]. This degeneration tends to progress with age and body mass index, particularly in the elderly population, where notable reductions in erector spinae CSA and increased fat content are observed [40,41]. Furthermore, these morphological abnormalities are closely associated with various spinal conditions, including lumbar disk herniation, lower back pain, and lumbar spinal stenosis, highlighting their relevance as diagnostic markers for spinal degeneration [42,43,44,45,46,47,48,49,50,51].

### 4.2. Surface Electromyography (sEMG)

**Figure 1:**
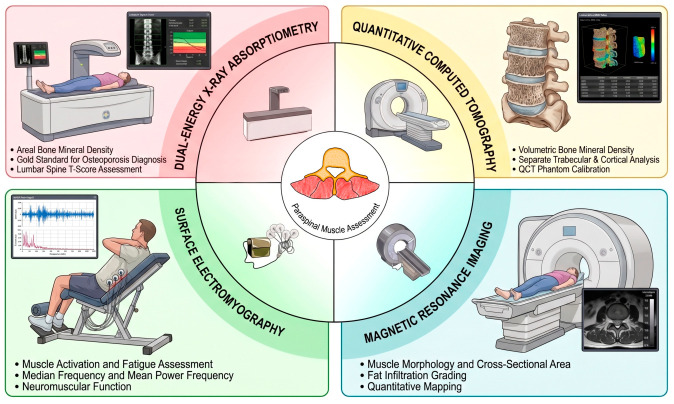
The assessment methods of bone mineral density and paraspinal muscles.

Surface electromyography (sEMG) is an effective method for dynamically assessing paraspinal muscle function. Time-domain parameters, such as Integrated Electromyogram (IEMG) and Root Mean Square (RMS), are utilized clinically to evaluate muscle strength production, activation levels, and localized fatigue. Frequency-domain analyses, primarily Mean Power Frequency (MPF) and Median Frequency (MF), offer insights into muscle fiber type distribution and spectral characteristics during exertion [52,53,54]. In the context of the muscle–bone unit, sEMG provides critical functional data that complements structural imaging, allowing for the quantification of actual muscle activation and the mechanical forces exerted on the lumbar spine during movement [55,56] (Figure 1).

## 5. Bone Mineral Density and Paraspinal Muscles

There is an association between decreased bone density and muscle function. (Table 1). The conceptualization of the spine not merely as an isolated skeletal structure but as a highly integrated “muscle–bone unit” is essential for understanding the progression of spinal degeneration. Within this framework, a unit of bone mass corresponds to a proportional amount of muscle mass, indicating that the mechanical load imposed on trabecular bone is directly modulated by surrounding musculature [57]. When paraspinal muscle function deteriorates, the dynamic stability of the spine is compromised, altering the stress distribution across the vertebral bodies.

### 5.1. The Association Between FI, CSA and Bone Loss

A substantial consensus across multiple imaging modalities indicates that adipose infiltration within the paraspinal muscles is a reliable indicator of concomitant bone mineral density decline. Rather than analyzing these findings in isolation, synthesizing the data from QCT, dual-energy CT (DECT), and MRI reveals a definitive pathophysiological pattern. Multiple independent cohorts have confirmed that increased FI in the paraspinal muscles, particularly the multifidus and erector spinae, is significantly and negatively correlated with lumbar vertebral BMD [58,59,60]. This lipomatous degeneration reflects a profound decline in muscle quality. Comprehensive analyses of large community-based populations consistently demonstrate that as intramuscular fat increases, trabecular volumetric BMD decreases accordingly [59,61]. This relationship persists regardless of the specific imaging technique utilized, whether it is chemical shift encoding-based water-fat MRI quantifying the proton density fat fraction (PDFF) or CT-based attenuation measurements [58,62]. Furthermore, demographic variables influence this progression, with older females exhibiting higher degrees of both FI and concurrent bone loss compared to their male counterparts [61].

The underlying mechanisms for this synchronous deterioration are fundamentally driven by active biomechanical and biochemical muscle–bone crosstalk, rather than isolated tissue aging [63]. Biomechanically, paraspinal muscle degeneration diminishes the essential mechanical loading exerted on the adjacent lumbar vertebrae. This reduction in mechanical stress disrupts normal mechanotransduction, which in turn diminishes osteoblastic bone formation and accelerates osteoclastic bone resorption. Biochemically, intermuscular adipose tissue functions as an active endocrine organ. The infiltrated fat secretes pro-inflammatory adipokines and alters the local myokine signaling profile, fostering a lipotoxic microenvironment. These locally acting paracrine factors directly disrupt bone homeostasis by stimulating osteoclastogenesis and inhibiting osteoblast function, thereby exacerbating trabecular bone loss [64]. Additionally, structural parameters such as the vertebral bone quality score, which independently predicts BMD, exhibit a strong positive correlation with the degree of paraspinal muscle degeneration [65]. Although the vast majority of the literature supports this negative correlation, isolated studies have reported null findings regarding the association between psoas fat content and BMD, indicating that the multifidus and erector spinae may be more biomechanically linked to the lumbar vertebrae than the anterior psoas muscle [66].

Unlike the uniform consensus regarding muscle quality, the relationship between muscle quantity, represented by the CSA and lumbar BMD remains contradictory. Some studies established a positive linear association between paraspinal muscle CSA and lumbar BMD. Quantitative measurements utilizing QCT and MDCT have demonstrated that larger CSA in the multifidus, erector spinae, and psoas major muscles moderately correlates with higher bone density in both osteopenic and normal populations [67,68,69,70]. These findings suggest that maintaining muscle bulk directly preserves the mechanical forces exerted on the spine, thereby stimulating osteoblast activity and maintaining bone mass.

Conversely, other studies failed to replicate these positive correlations, particularly when assessing populations with existing spinal pathologies. In patients undergoing lumbar spine fusion surgery or those diagnosed with severe lumbar spinal stenosis, researchers found no significant association between paraspinal muscle CSA and vertebral BMD [71,72]. The discrepancy in these findings can be explained by several confounding factors. First, in pathological states such as severe disc degeneration or stenosis, the local anatomical environment is altered, and patients often alter their biomechanical loading patterns due to chronic pain. Second, a larger anatomical CSA does not necessarily equate to a larger functional CSA. A muscle may retain a large overall volume while being heavily infiltrated by non-contractile adipose and fibrotic tissue. Therefore, measuring total CSA without adjusting for fat content may overestimate functional muscle capacity, obscuring its true mechanical relationship with bone density.

### 5.2. Predictive Value for Fractures and Surgical Outcomes

Integrating paraspinal muscle parameters into the clinical evaluation of bone health dramatically enhances fracture risk stratification. Treating BMD and muscle degeneration as independent variables underestimates the synergistic failure of the muscle–bone unit.

Recent advancements in prognostic modeling have demonstrated that appending paraspinal muscle characteristics and trabecular texture to traditional prediction models identifies incident vertebral fractures significantly better than clinical parameters like age, BMI, and BMD alone [73]. The mechanical stability of the spine relies heavily on the “Bone Load Index,” and the interaction between this index and local fat infiltration serves as a powerful independent risk factor for primary vertebral fractures [74]. Furthermore, patients with multiple fractures may present with a larger total psoas CSA, but their functional, fat-free muscle area is severely diminished [75]. By mathematically synthesizing these variables, clinical nomograms have been developed that achieve high predictive accuracy for osteoporotic vertebral compression fractures [76].

The functional status of paraspinal muscles is also emerging as a critical determinant of postoperative outcomes. Following surgical interventions such as percutaneous vertebroplasty or kyphoplasty, the biomechanics of the augmented vertebra are permanently altered. High fat infiltration rates in the paraspinal muscles significantly impair the spine’s ability to absorb and distribute mechanical stress. Consequently, extensive FI has been identified across multiple independent studies as the most robust independent predictor for new, secondary vertebral compression fractures adjacent to the surgical site [77,78,79].

### 5.3. The Impact of Mechanical Loading

The functional deterioration of the muscle–bone unit is not entirely irreversible. Mechanical stress remains a primary biological driver for preserving trabecular bone mass in localized anatomical regions [80,81]. While cross-sectional observations confirm that intentional, generalized exercise may not be sufficient to reverse established negative correlations between FI and BMD [82], targeted biomechanical interventions yield measurable structural improvements. Multi-component exercise programs specifically designed for the lumbar region effectively enhance paraspinal muscle CSA and increase lumbar spine trabecular BMD simultaneously over extended periods [83]. The fundamental mechanism behind exercise-induced bone preservation is governed by direct force transmission. Combined high-impact and resistance exercises generated paraspinal muscle forces 1.22 to 8.18 times greater than daily activities like walking. Therefore, reversing the degeneration of the muscle–bone unit requires high-intensity, site-specific mechanical loading rather than simply increasing overall aerobic expenditure [84].

**Table 1:** The studies about paraspinal muscles and BMD.

Years	Researchers	Samples	Research Method	Conclusions	Research Type
2025	Liu et al. [84]	10	QCT	Paravertebral muscle forces and intervertebral compression forces were the main reasons for the improvement of spine BMD.	NRCT
2025	Hummel et al. [73]	843	DXA and QCT	Adding paraspinal muscle parameters to prediction models improved the identification of incident vertebral fractures.	Retrospective study
2025	Jiang et al. [74]	144	QCT	Psoas major FI served as an independent risk factor for vertebral fractures.	Retrospective study
2025	Sönmez et al. [75]	77	MRI	The functional CSA was significantly reduced in the context of multiple fractures.	Retrospective study
2025	Wang et al. [76]	260	MRI and QCT	Developing a comprehensive nomogram that includes BMD and muscle FI.	Retrospective study
2025	Yang et al. [78]	250	MRI	High FI rates in the psoas and erector spinae muscles are significant risk factors for secondary vertebral fractures.	Prospective study
2025	Zhuo et al. [79]	150	DXA and MRI	Paraspinal FI is a critical biomarker for refracture risk.	Retrospective study
2024	Tang et al. [77]	288	MRI	High FI rates in the psoas and erector spinae muscles are significant risk factors for secondary vertebral fractures.	Retrospective study
2022	Li et al. [59]	367	QCT	Paraspinal muscle FI was negatively correlated with spine BMD.	Prospective study
2022	Zhou et al. [60]	119	DECT and QCT	The paraspinal muscles’ fat content had a fairly significant inverse association with lumbar BMD.	Retrospective study
2022	Yang et al. [61]	605	MRI and QCT	Paraspinal muscle FI but not muscle CSA increased with age in both sexes and was related to lower lumbar spine vBMD.	Retrospective study
2022	Li et al. [67]	367	QCT	Paraspinal muscle FI was negatively correlated with spine BMD.	Prospective study
2022	Tekin et al. [68]	90	CT and DXA	The most significantly associated parameters with lumbar spine BMD were psoas and erector spinae muscles’ CSA and index values.	Retrospective study
2022	Touban et al. [70]	60	CT and DXA	This study demonstrated a significant positive association between central muscle CSA and BMD.	Retrospective study
2022	Chiapparelli et al. [71]	105	MRI and QCT	In females, a significant positive correlation existed between the FCSA and vBMD of the lumbar paraspinal muscles at different lumbosacral levels, which was not observed in males.	Retrospective study
2022	Han et al. [72]	38	DXA and QCT	Paraspinal muscle morphology had a stronger correlation with lumbar BMD measured by CT than by DXA.	Retrospective study
2022	Gurusamy et al. [82]	1923	CT	Paraspinal muscle density was positively associated with higher lumbar BMD.	Retrospective study
2020	Sollmann et al. [69]	116	CT	There were level-dependent interactions between paraspinal muscle characteristics and lumbar BMD.	Retrospective study
2020	Turcotte et al. [83]	180	QCT	Changes in paraspinal muscle size were associated with the changes in spinal BMD, independent of exercise.	RCT
2019	Zhao et al. [58]	88	MRI and QCT	Paraspinal muscle FI was negatively correlated with spine BMD.	Prospective study
2019	Ekin et al. [62]	312	CT	A strong association between reduced BMD and muscle atrophy was found.	Retrospective study

Note: BMD: bone mineral density, CSA: cross-sectional surface area, CT: computed tomography, DECT: dual-energy computed tomography, DXA: dual X-ray absorptiometry, FCSA:Functional cross-sectional surface area, FI: fat infiltration, MRI: magnetic resonance imaging, NRCT: non-randomized controlled trial, QCT: quantitative computed tomography, RCT: randomized controlled trial, vBMD: volumetric bone mineral density.

## 6. Conclusions

This review highlights the growing evidence supporting a critical synergy between paraspinal muscle status and BMD. Current synthesized evidence demonstrates that paraspinal muscle fat infiltration is a remarkably consistent and independent marker for trabecular bone loss and elevated fracture susceptibility. While the role of gross muscle volume remains context-dependent and heavily influenced by functional muscle quality, the integration of quantitative muscle parameters with traditional BMD measurements substantially improves clinical risk stratification. However, current research is limited by its predominant reliance on retrospective data and inconsistent assessment protocols. Future directions should prioritize large-scale prospective cohort studies to elucidate causal mechanisms and randomized controlled trials to validate exercise interventions based on biomechanical loading. Ultimately, establishing a standardized assessment system for the “muscle–bone unit” is essential for advancing precision medicine and holistic osteoporosis management.

## Data Availability

Not applicable.

## Abbreviations

BMD: bone mineral density, CSA: cross-sectional area, DXA: dual-energy x-ray absorptiometry, QCT: quantitative computed tomography, sEMG: surface electromyography, IEMG: integrated electromyogram, RMS: root mean square, MPF: mean power frequency, MF: median frequency, MDCT: multi-detector computed tomography.
